# Ethanolic Extract of Propolis and CAPE as Cardioprotective Agents against LPS and IFN-α Stressed Cardiovascular Injury

**DOI:** 10.3390/nu16050627

**Published:** 2024-02-23

**Authors:** Anna Kurek-Górecka, Małgorzata Kłósek, Grażyna Pietsz, Radosław Balwierz, Paweł Olczyk, Zenon P. Czuba

**Affiliations:** 1 Department of Community Pharmacy, Faculty of Pharmaceutical Sciences in Sosnowiec, Medical University of Silesia in Katowice, Kasztanowa 3, 41-200 Sosnowiec, Poland; polczyk@sum.edu.pl; 2 Department of Microbiology and Immunology, Faculty of Medical Sciences in Zabrze, Medical University of Silesia in Katowice, Jordana 19, 41-808 Zabrze, Poland; gpietsz@sum.edu.pl (G.P.); zczuba@sum.edu.pl (Z.P.C.); 3 Institute of Chemistry, University of Opole, Oleska 48, 45-052 Opole, Poland; radoslaw.balwierz@uni.opole.pl

**Keywords:** caffeic acid phenethyl ester, propolis, cytokines, adhesion molecules, inflammation

## Abstract

The inflammatory process is triggered by several factors such as toxins, pathogens, and damaged cells, promoting inflammation in various systems, including the cardiovascular system, leading to heart failure. The link between periodontitis as a chronic inflammatory disease and cardiovascular disease is confirmed. Propolis and its major component, caffeic acid phenethyl ester (CAPE), exhibit protective mechanisms and anti-inflammatory effects on the cardiovascular system. The objective of the conducted study was to assess the anti-inflammatory effects of the Polish ethanolic extract of propolis (EEP) and its major component—CAPE—in interferon-alpha (IFN-α), lipopolysaccharide (LPS), LPS + IFN-α-induced human gingival fibroblasts (HGF-1). EEP and CAPE were used at 10–100 µg/mL. A multiplex assay was used for interleukin and adhesive molecule detection. Our results demonstrate that EEP, at a concentration of 25 µg/mL, decreases pro-inflammatory cytokine IL-6 in LPS-induced HGF-1. At the same concentration, EEP increases the level of anti-inflammatory cytokine IL-10 in LPS + IFN-α-induced HGF-1. In the case of CAPE, IL-6 in LPS and LPS + IFN-α induced HGF-1 was decreased in all concentrations. However, in the case of IL-10, CAPE causes the highest increase at 50 µg/mL in IFN-α induced HGF-1. Regarding the impact of EEP on adhesion molecules, there was a noticeable reduction of E-selectin by EEP at 25, 50, and100 µg/mL in IFN-α -induced HGF-1. In a range of 10–100 µg/mL, EEP decreased endothelin-1 (ET-1) during all stimulations. CAPE statistically significantly decreases the level of ET-1 at 25–100 µg/mL in IFN-α and LPS + IFN-α. In the case of intercellular adhesion molecule-1 (ICAM-1), EEP and CAPE downregulated its expression in a non-statistically significant manner. Based on the obtained results, EEP and CAPE may generate beneficial cardiovascular effects by influencing selected factors. EEP and CAPE exert an impact on cytokines in a dose-dependent manner.

## 1. Introduction

Periodontitis and other oral cavity infections may increase the risk of cardiovascular diseases by affecting the concentration of coagulation and fibrinolytic factors. Periodontal disease is associated with an increased risk of coronary artery disease, myocardial infarction, ischemic heart disease, and stroke [1,2]. Periodontal disease is caused by bacterial infection of the gums, periodontium, adjacent alveolar bone, and root cement. Among the bacteria which are responsible for inflammation in periodontal tissue are *Porphyromonas gingivalis*, *Treponema denticola*, *Tannarella forsythia*, *Bacteroides forsythus*, *Aggregatibacter actinomycetemcommitans*, *Prevotella intermedia*, *Streptococcus sanguis*, and *Fusobacterium nucleatum* [2,3]. Bacteria and their products present in the biofilm stimulate immunocompetent cells to produce and release inflammatory mediators. Bacteria, their metabolic products, attach to the tissues, and mediators of the immune reaction can enter the bloodstream. Additionally, during inflammation, metalloproteinases, prostaglandins, eicosanoids, kinins, cytokines, chemokines, and complement activation products are transferred to the blood circulatory system.

Among the mediators that play an important role in the pathomechanism of periodontitis and cardiovascular diseases are interleukins (IL). Pro-inflammatory interleukins influence the increased expression of adhesion molecules, which are predictive factors for coronary artery disease [4,5,6,7].

An LPS-induced cellular inflammation model and IFN-α-induced side effects of the cardiovascular system were used. IFN-α is used in treating patients suffering from hepatitis B and C, lymphoma (lymph node cancer), malignant melanoma (skin cancer), genital warts, hairy cell leukaemia (blood cell cancer), and Kaposi sarcoma (AIDS-related tumor). The mentioned treatment causes common side effects, including hypertension, palpitations, and tachycardia. Sometimes, it may cause hypotension, cardiomyopathy, or peripheral ischemia [8]. In sporadic cases were observed angina pectoris, arrhythmia, atrial fibrillation, bradycardia, cardiac failure, cardiac ischemia, cyanosis, extrasystoles, myocardial infarction, postural hypotension, and thrombophlebitis. Cardiovascular side effects, especially arrhythmia, appeared to be associated with preexisting cardiovascular disease and prior use of cardiotoxic agents.

Propolis and its component—CAPE—exhibit potential beneficial cardiovascular effect [8,9]. The cardiovascular effect of propolis is connected with its anti-atherosclerotic, anti-hemostatic, antihypertensive, anti-angiogenesis, endothelial-protecting, myocardial-protecting, antioxidant, and anti-inflammatory activities [10,11]. Therefore, propolis may be a natural cardioprotective agent, which counteracts inflammation during cardiovascular diseases. The study evaluated the anti-inflammatory impact of Polish ethanolic extract of propolis (EEP) and CAPE on HGF-1 stimulated with lipopolysaccharide (LPS) and interferon-α (IFN-α) using the cellular inflammation model. IFN-α belongs to the type I interferons, which are pluripotent cytokines secreted by most cell types in response to viral infections. Moreover, IFN-α enhances LPS-mediated responses. Parra-Izquierdo I. et al. demonstrated cooperation between IFN-α, as well as LPS, on the induction of adhesion molecules [12]. The release of selected cytokine IL-6 and adhesion factors such as ICAM-1, E-selectin, and protein ET-1 by the HGF-1 cell line treated with EEP and CAPE was determined in the culture supernatant. The mentioned factors are modern markers of heart failure [5,13,14,15,16,17]. Moreover, we investigated the impact of EEP and CAPE on the release of anti-inflammatory cytokine—IL-10. It has been hypothesized that high expression of pro-inflammatory cytokines and adhesion molecules may be a predictor of coronary artery disease, as increased expression of anti-inflammatory cytokines occurs in patients with coronary artery disease [14]. The novelty of these studies is related to the lack of reports regarding the impact of EEP on the secretion of heart failure markers by HGF-1.

## 2. Materials and Methods

The sample of crude propolis was obtained from an ecological area in the south of Poland. CAPE of synthetic origin was purchased from Sigma Aldrich (Munich, Germany). Ethanol 96% was purchased from POCH (Gliwice, Poland). DMSO was obtained from Sigma Chemical Company (St. Louis, MO, USA). LPS, as a component of the cell wall of Gram-negative bacteria, was obtained from *Escherichia coli* O26:B6. It was purified by trichloroacetic acid extraction. LPS was purchased from Fluka (Sigma-Aldrich, Saint Louis, MO, USA). IFN-α was biosynthetically generated and constitutes the product of a cloned human leukocyte interferon gene inserted into *Escherichia coli*; it was purchased from Roche Company (Warsaw, Poland). HGF-1 was obtained from the American Type Culture Collection (ATCC, Manassas, VA, USA). Dulbecco’s Modified Eagle’s Medium (DMEM), Fetal Bovine Serum (FBS), and Trypsin-EDTA were purchased from Sigma Aldrich (Darmstadt, Germany). Penicillin-Streptomycin solution (10,000 U/mL Penicillin, 10 mg/mL Streptomycin) was purchased from PAN Biotech (Aidenbach, Germany). 3-(4,5-dimethyl-2-thiazyl)-2,5 diphenyltetrazolium (MTT) was provided by Sigma Chemical Company (St. Louis, MO, USA). The Bio-Plex Pro Human Cytokine Kit assay was obtained from R & D Systems Inc. (Minneapolis, MN, USA).

### 2.1. Preparation of EEP

The preparation of ethanol extract of propolis has been previously described by Kurek-Górecka et al. [18]. The solid ethanolic extract of propolis was obtained in a two-step extraction process. The raw propolis (100 g) was added to ethanol 70% (1000 mL), and this mixture was stirred for 24 h. Next, the mixture was filtered (vacuum filtration). The sediment after filtration was collected and again mixed with ethanol 70% (500 mL); then, the mixture was stirred for 24 h, and the first filtrate obtained after filtration was stored. The mixture was then filtered (vacuum filtration), and the second filtrate was joined with the first. The total extract (first and second filtrates joined) was concentrated on the vacuum rotary evaporator and dried to a solid in the vacuum oven. For further examinations, the solid ethanolic extract of propolis was diluted in DMSO at concentrations of 10, 25, 50, and 100 µg/mL.

The extraction efficiency was calculated by dividing the mass of the solid extract obtained by the used mass of the raw propolis and expressed as a percentage. The extraction efficiency was 54%.

### 2.2. HGF-1 Cell Culture

HGF-1, which was isolated from healthy human gingiva, was provided by the American Type Culture Collection (ATCC, Manassas, VA, USA). The cultivation of HGF-1 was performed at 37 °C under sterile conditions. The medium growth was Dulbecco’s Modified Eagle’s Medium (DMEM), supplemented with 10% fetal bovine serum (FBS). Moreover, penicillin/streptomycin solution (final concentrations of 100 U/mL and 100 µg/mL, respectively) was added to the medium. The medium was equilibrated for use with 5% CO_2_/95% air in the incubator. Trypsin-EDTA at a concentration of 0.05% was used to detach the cells. To determine the number of cells, Bürker’s counting chamber was used. The cells were calculated using the formula:

Number of cells in 1 mL = (4 squares counted: 2) × 100 × 1000

### 2.3. LPS, IFN-α, LPS + IFN-α Stimulation of HGF-1 and EEP or CAPE Treatments

The HGF-1 fibroblasts were incubated with LPS (final concentration: 200 ng/mL), IFN-α (final concentration 100 U/mL), and combined LPS+ IFN-α for 24 h in 96-well plates. EEP and CAPE were used in this experiment at concentrations of 10, 25, 50, and 100 µg/mL.

All analyses were carried out in triplicate, including positive and negative controls: the fibroblasts activated with LPS, IFN-α or combined LPS+ IFN-α without EEP or CAPE treatment, and the native fibroblasts (no stimulation or treatment added to the culture medium), respectively.

### 2.4. Cell Viability Assay

The MTT assay, as a colorimetric assay for measuring cell viability, was used. This assay is based on the reduction of a yellow 3-(4,5-dimethyl-2-thiazyl)-2,5-diphenyl-2H-tetrazolium bromide to a blue formazan crystal by viable cells. The insoluble formazan crystals were dissolved using a DMSO, and the results were estimated by measuring the absorbance at 550 nm. In the MTT assay, EEP and CAPE were used at concentrations of 10, 25, 50, and 100 µg/mL, with or without LPS and IFN-α, as well as combined EEP + IFN-α. The time of incubation of the cell lines with the MTT salt was 4 h. The results were calculated based on the absorbance of the sample and control. The measured outcomes were calculated from the formula:

% cell viability = sample absorbance 100/absorbance of the control

### 2.5. Multiplex Bead-Based Cytokine and Adhesive Molecules Assay

Bio-Plex carboxylated magnetic beads were used during the multiplex assay. These beads were internally labeled with two fluorescent dyes for bead identification. The Bio-Plex Human cytokine kit (R & D Systems Inc., Minneapolis, MN, USA) was used, according to the producer’s instruction, to determine the selected cytokines IL-6 and IL-10 and adhesion factors such as ICAM-1, E-selectin, and ET-1 in the supernatant of HGF-1. In IFN-α, LPS and the combined IFN-α + LPS-induced fibroblasts HGF-1 were incubated with and without EEP, as well as CAPE, at concentrations of 10, 25, 50, and 100 µg/mL for 24 h. The concentrations of IL-6, Il-10, E-selectin, ICAM, and ET-1 released from HGF-1 were evaluated 24 h after treatment with EEP and CAPE. A standard dilution series and a blank were prepared using the kit-supplied references of selected cytokines and adhesion factors. The supernatant obtained from the HGF-1 culture line was added to a special 96-well plates, which was then incubated with antibody-conjugated magnetic beads for 120 min and washed with buffer. The ELx50 magnetic washer (BioTek, Winooski, VT, USA) was used. Then, biotinylated detection antibodies were added to each well, and the samples were incubated for 60 min and washed with buffer. Next, streptavidin–phycoerythrin conjugates were added to each well, and the samples were incubated for 30 min. In the next step, unbounded streptavidin was removed by washing with buffer. And finally, selected cytokines and adhesion molecules bounded with beads were determined in the Bio-Plex Array Reader (Bio-Plex 200 System). The fluorescence was assayed using Bio-Plex Manager software, Version 5.0 (BIO-RAD, Hercules, CA, USA).

### 2.6. Statistical Analysis

All determinations were carried out, and the obtained results were expressed as means. STATISTICA 13.1 software (StatSoft Inc., Tulsa, OK, USA) was used for analysis. To compare the effect of EEP and CAPE at different concentrations on IL-6, IL-10, E-selectin, ET-1, and ICAM-1 secretion by HGF-1 fibroblasts and stimulated by LPS, IFN-α, and LPS + IFN-α, we applied one-way ANOVA. Statistical significance was calculated using Fisher’s LSD test. Statistical significance was considered when *p* < 0.05. Moreover, the results were analyzed using HCA and PCA. The HCA was performed with full linkage using Euclidean distance. The PCA model was estimated using the NIPALS iterative algorithm.

## 3. Results

The conducted study was designed to evaluate the impact of EEP and its isolated compound—CAPE—on the concentration of pro-inflammatory and anti-inflammatory cytokines, as well as on the adhesion factors, whose intensive production is associated with heart failure. The composition of the solid ethanol extract of Polish propolis has been previously examined and described by Kurek-Górecka et al. [18].

In the proposed concentrations, EEP and CAPE did not exert a cytotoxic effect on HGF-1 fibroblasts, which has been confirmed previously [18]. Hence, EEP and CAPE were used at a range of 10–100 µg/mL for further studies. Our research is a continuation of studies on the anti-inflammatory effect of propolis.

### 3.1. Effect of EEP on IL-6, IL-10, E-Selectin, ET-1, and ICAM-1 Secretion in LPS, IFN-α, and LPS + IFN-α-Induced HGF-1

**Figure 1:**
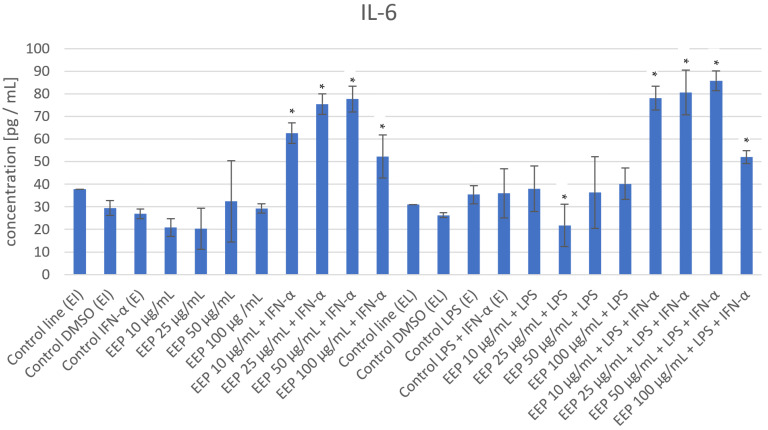

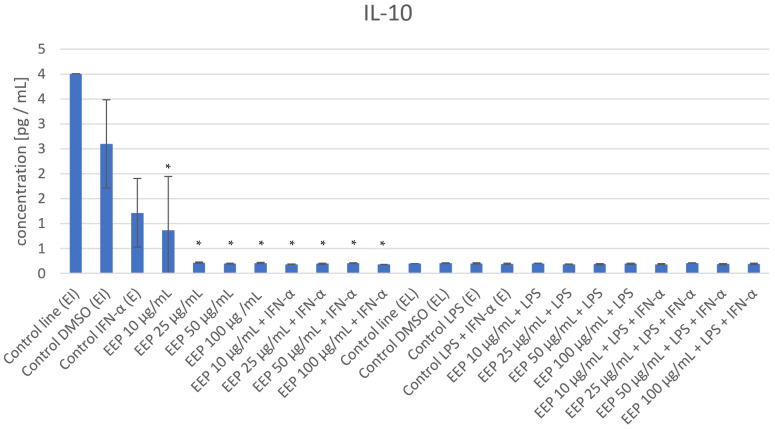

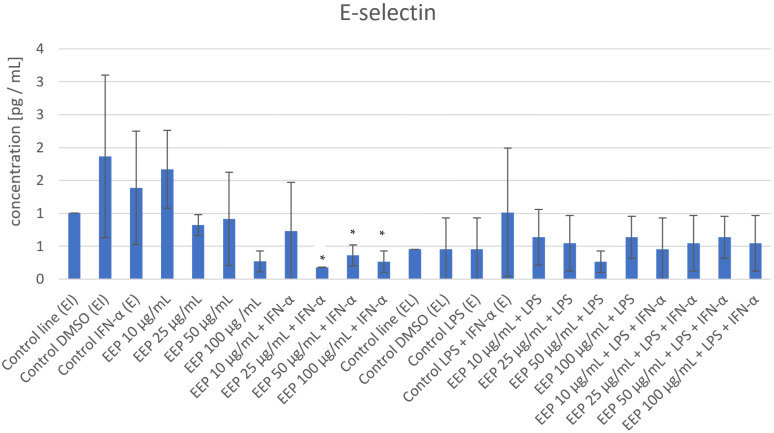

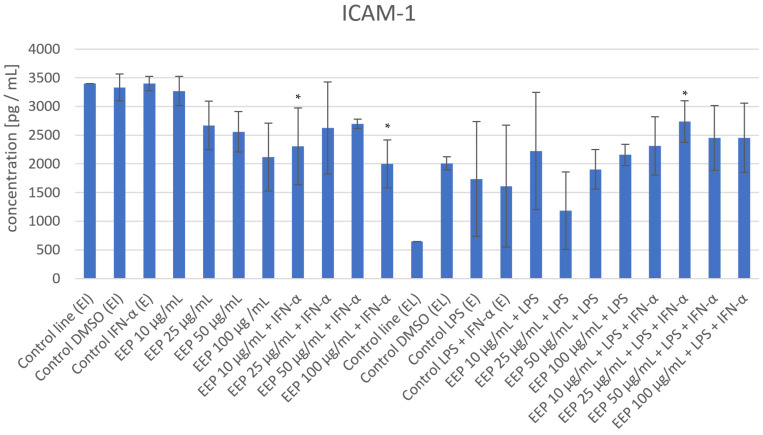

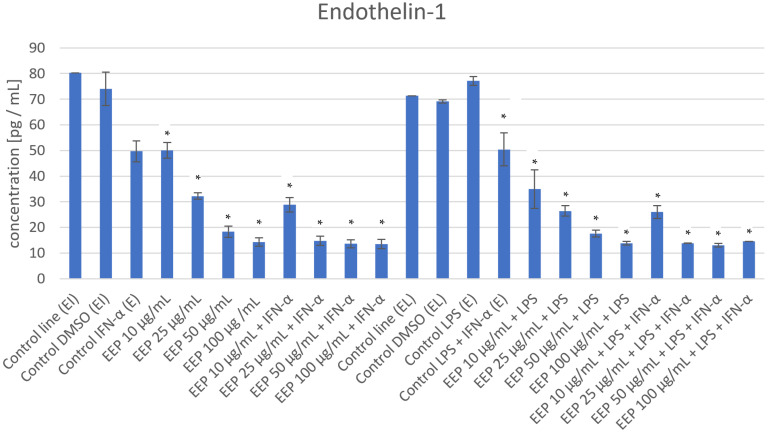
The effect of EEP on the secretion of selected interleukins and adhesion molecules in LPS, IFN-α, and LPS + IFN-α-induced HGF-1. * means *p* < 0.05.

The impact of EEP on the production of selected factors was assayed via experimental conditions in LPS, IFN-α, and combined LPS + IFN-α–induced HGF-1 cells, and the results are presented in Figure 1 and Table 1. According to data from the literature, it was expected that LPS, IFN-α, and combined LPS + IFN-α may increase the levels of the evaluated secretion factors noted in the control cell line [12,18].

In the LPS-induced HGF-1 cells, EEP at a concentration of 25 µg/mL decreased the production of IL-6 (*p* = 0.0459). In other concentrations, an elevation in IL-6 secretion was noted; therefore, EEP (25 µg/mL) reduced IL-6 in a dose-dependent manner. During the determination of IL-10 expression, EEP at all analyzed concentrations (10, 25, 50, and 100 µg/mL) caused the reduction of IL-10 levels following IFN-α stimulation. The variations achieved statistical significance (*p* = 0.0006, *p* = 0.0007, *p* = 0.0007, *p* = 0.0005, respectively). A similar pattern was observed following LPS stimulation, as EEP at concentrations of 25, 50 and 100 µg/mL decreased the IL-10 level; however, the variations did not achieve statistical significance (Table 1). In the case of stimulation of HGF-1 following treatment with combined LPS + IFN-α EEP at a concentration of 10 µg/mL, a reduction of the IL-10 level was observed, but without statistical significance (Table 1). However, at a concentration of 25 µg/mL, the effect was noticeably increased. The impact of EEP treatment at all analyzed concentrations (10–100 µg/mL) resulted in the decrease in E-selectin following IFN-α stimulation. At concentrations of 25, 50, and 100 µg/mL of EEP, statistically significant differences were observed (*p* = 0.01, *p* = 0.027, *p* = 0.0164, respectively). A similar pattern was observed following combined LPS + IFN-α stimulation. However, the observed differences were not statistically significant. Regarding the impact of EEP treatment on ICAM-1, a noticeable reduction of this adhesion molecule was observed in IFN-α-induced HGF-1. At concentrations of 10 and 100 µg/mL, statistically significant differences were observed (*p* = 0.0247, *p* = 0.0048, respectively).

The behavior of ET-1 was similar to that of IL-10. In IFN-α, LPS, as well as combined LPS + IFN-α-induced HGF-1, EEP at all analyzed concentrations markedly decreased the levels of ET-1. However, at a concentration of 10 µg/mL EEP treatment following IFN-α stimulation resulted in variations that achieved statistical significance (*p* = 0.0).

### 3.2. Effect of CAPE on IL-6, IL-10, E-Selectin, ET-1, and ICAM-1 Secretion in LPS, IFN-α, LPS + IFN-α-Induced HGF-1

**Figure 2:**
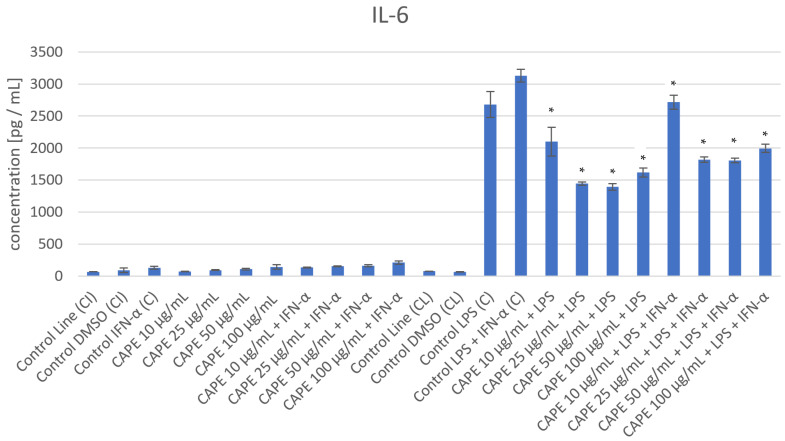

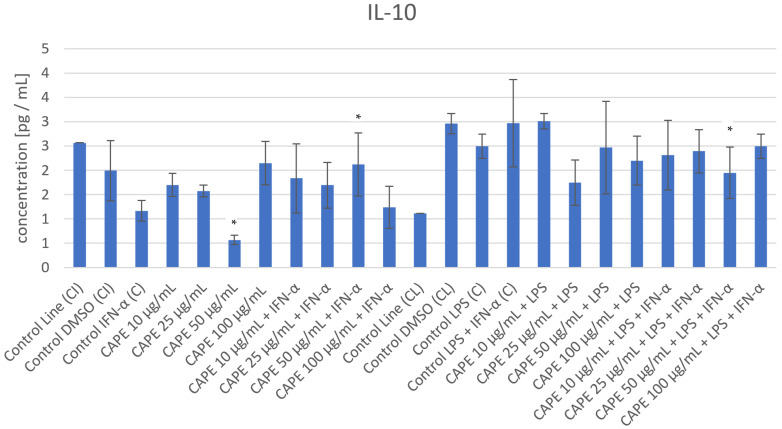

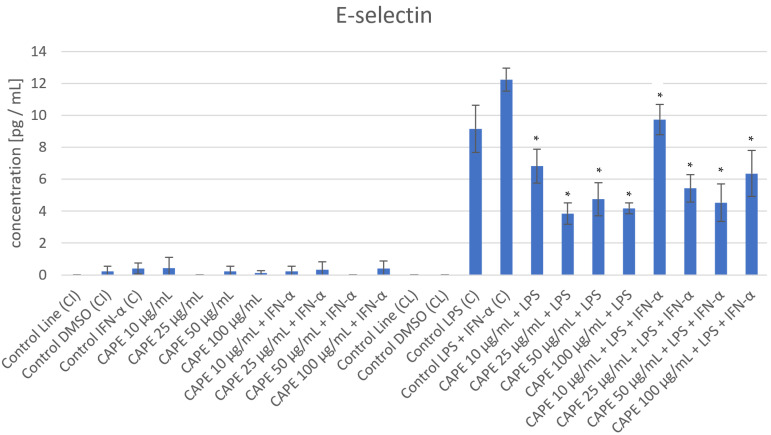

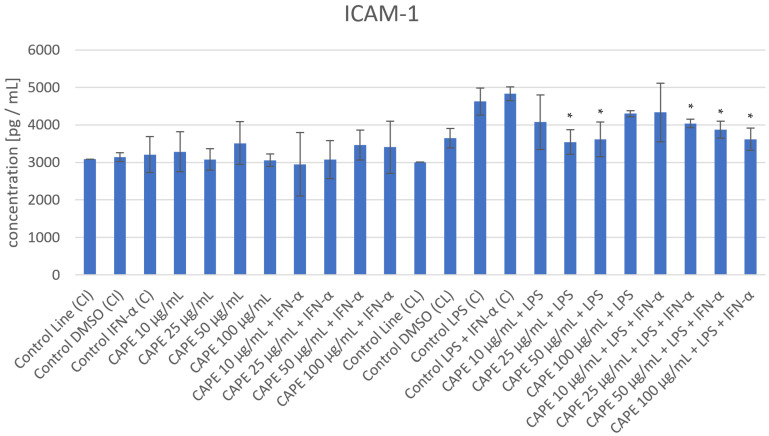

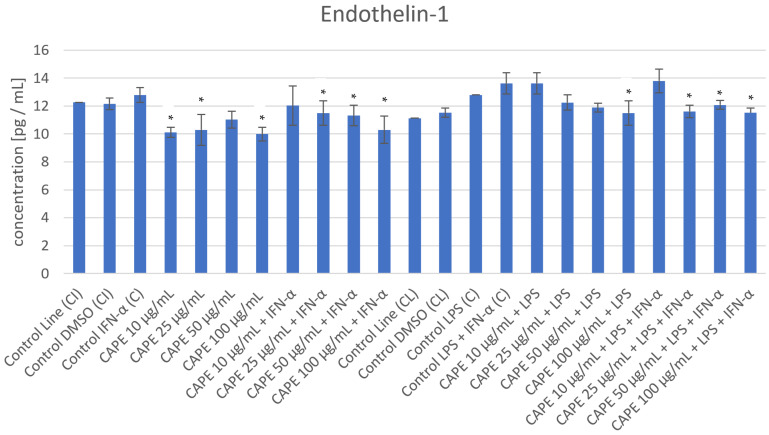
The effect of CAPE on the secretion of selected interleukins and adhesion molecules in LPS, IFN-α, and LPS + IFN-α-induced HGF-1. * means *p* < 0.05.

The impact of CAPE on the production of selected factors was assayed via experimental conditions in LPS, IFN-α, as well as combined LPS + IFN-α-induced HGF-1 cells, and the results are presented in Figure 2 and Table 2. In HGF-1 fibroblast cells following LPS, as well as combined LPS+ IFN-α stimulation, CAPE reduced IL-6 at all analyzed concentrations (10–100 µg/mL), and the variations achieved statistical significance (*p* = 0.0). However, in the case of HGF-1 stimulation with IFN-α, CAPE, in all analyzed concentrations, increased the level of IL-6 (Table 2). In the context of the impact of CAPE on the production of IL-10, a decrease in the level of IL-10 was observed following LPS + IFN-α treatment at all analyzed concentrations, while subsequent LPS stimulation resulted in a decrease in IL-10 observed at 25, 50, and 100 µg/mL. The obtained results did not achieve statistical significance, except in the case of CAPE at 50 µg/mL in LPS + IFN-α-induced HGF-1 (*p* = 0.0173) (Table 2). However, we noticed an increase in IL-10 following IFN-α exposure at all analyzed concentrations of CAPE. The highest increased level of IL-10 was noticed at 50 µg/mL of CAPE, which achieved statistical significance (*p* = 0.0253). Regarding the impact of CAPE on the adhesion factors, it is worth noting that CAPE caused a decreased production of E-selectin following IFN-α stimulation at concentrations of 10, 25, and 50 µg/mL, without statistical significance, and decreased production of E-selectin in all analyzed concentrations following LPS as well as LPS + IFN-α stimulation. Regarding stimulation by LPS and combined LPS + IFN-α, the differences achieved statistical significance (*p* = 0.005, *p* = 0.0, *p* = 0.0, *p* = 0.0, respectively) for LPS stimulation, and (*p* = 0.0033, *p* = 0.0, *p* = 0.0, *p* = 0.0, respectively) for LPS + IFN-α stimulation. In the case of ICAM-1, the measured outcomes show that CAPE decreased the production of ICAM-1 at concentrations 10 and 25 µg/mL in IFN-α-induced HGF-1, while in LPS, as well as in combined LPS + IFN-α-induced HGF-1, stimulation caused a decrease at all analyzed concentrations (10–100 µg/mL). the measured outcomes achieved statistical significance in the case of LPS-induced HGF-1 treatment of CAPE at concentrations of 25 and 50 µg/mL (*p* = 0.0062, *p* = 0.0102, respectively) and in the case of LPS + IFN-α-induced HGF-1 treatment of CAPE at concentrations of 25, 50, and 100 µg/mL (*p* = 0.0403, *p* = 0.0138, *p* = 0.0024, respectively) (Table 2). In addition, CAPE reduced the production of ET-1 at all analyzed concentrations (10–100 µg/mL) following IFN-α treatment. At concentrations of 25, 50, and 100 µg/mL, CAPE induced statistically significant changes (*p* = 0.0294, *p* = 0.0135, *p*= 0.0001, respectively). However, in the case of post-stimulation by LPS, as well as combined LPS + IFN-α, the treatments decreased ET-1 in a range of 25–100 µg/mL by CAPE. The statistically significant variations were noticed in the concentration of CAPE at 100 µg/mL following LPS stimulation (*p* = 0.0287) as well as in the concentrations of CAPE at 25, 50, and 100 µg/mL following treatment with combined LPS + IFN-α (*p* = 0.001, *p* = 0.0094, *p* = 0.0006, respectively).

### 3.3. Comparative Effect of EEP and CAPE on IL-6, IL-10, E-Selectin, ET-1, and ICAM-1 Secretion in LPS, IFN-α, LPS + IFN-α-Induced HGF-1

**Figure 3:**
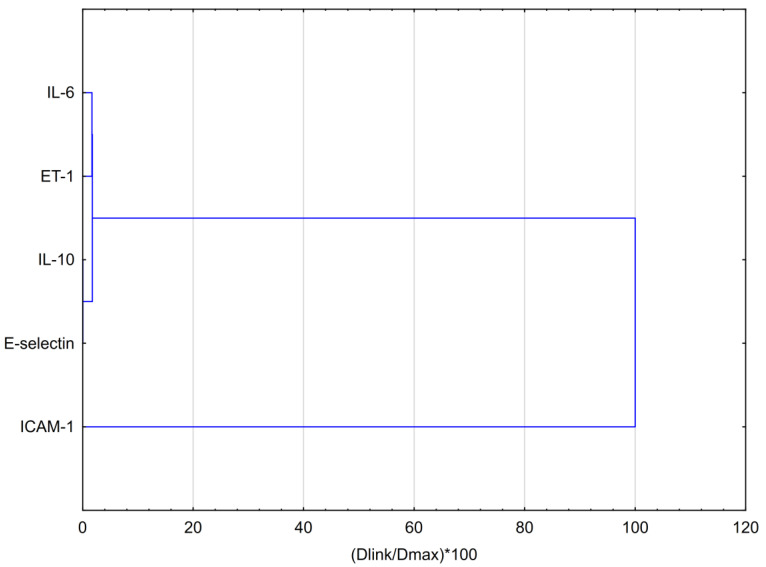
Dendrogram obtained via the HCA analysis of data regarding the behavior of IL-6, IL-10, E-selectin, ET-1, and ICAM-1 secretion by HGF-1 fibroblasts stimulated by LPS, IFN-α, and LPS + IFN-α following treatment with EEP.

To compare the impact of EEP and its major compound—CAPE—on the secretion of selected interleukins and adhesion molecules, hierarchical clustering analysis (HCA) and principal component analysis (PCA) were employed. The obtained results, analyzed by HCA for EEP, are depicted in Figure 3. Considering the Euclidean distances in analyzing the behavior of the evaluated markers for treatment with EEP, it is noticeable that all data could be clustered into three groups. The first cluster demonstrates the similar behavior of IL-6 and ET-1. It was noticed that EEP exerts a strong impact on the expression of IL-6 and ET-1. The second cluster indicates that IL-6 treated with E-selectin demonstrates comparable behavior to that of treatment with EEP. Different behavior is exhibited by ICAM-1, and this indicates that EEP exerts a disparate impact on this adhesion molecule.

**Figure 4:**
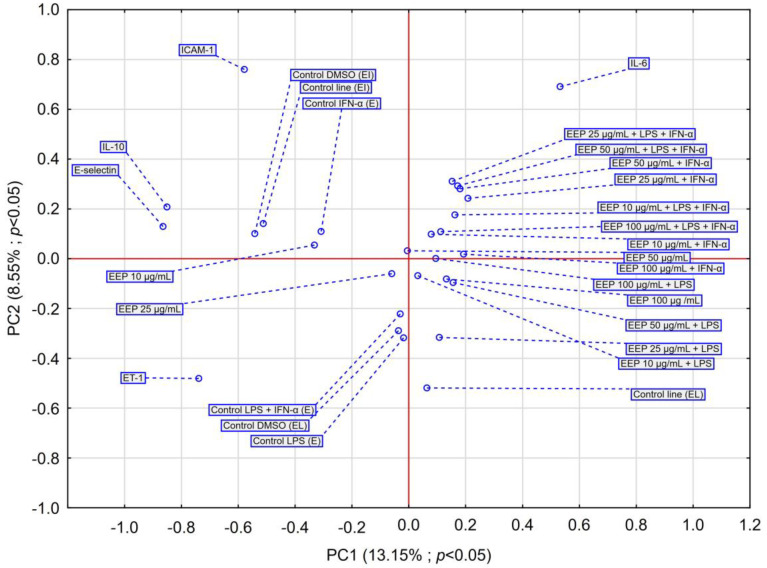
PCA score plot of obtained data based on the effect of EEP at different concentrations on IL-6, IL-10, E-selectin, ET-1, and ICAM-1 secretion by HGF-1 fibroblasts stimulated by LPS, IFN-α, and LPS + IFN-α.

The PCA score plots were prepared regarding the effect of EEP on selected markers, as shown in Figure 4. The insights from the PCA analysis of the EEP effect on selected markers are partially confirmed by the dendrogram obtained in the HCA analysis.

The conducted analysis indicates that IL-6 belongs toa group of markers on which EEP exerts a noticeable impact. IL-10 and E-selectin exhibit similar behaviors, whereas ICAM-1 expresses a different activity. However, ET-1 shows the most unique behavior.

**Figure 5:**
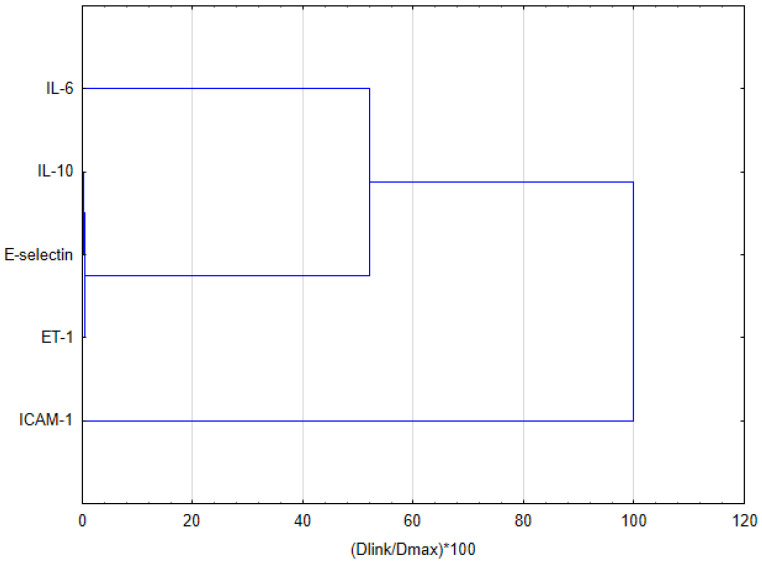
Dendrogram obtained via the HCA analysis of data regarding the behavior of IL-6, IL-10, E-selectin, ET-1, and ICAM-1 secretion by HGF-1 fibroblasts stimulated by LPS, IFN-α, and LPS + IFN-α following treatment with CAPE.

Regarding the impact of CAPE on selected markers based on HCA (Figure 5) analyzing Euclidean distances, it is noticeable that the impact on the cytokines of CAPE is disparate to the impact of EEP. It may be noted that IL-10 and E-selectin are included in the first cluster and exhibit similar behaviors following CAPE treatment. The second cluster involves IL-10, E-selectin, and ET-1. Therefore, CAPE exerts a similar impact on the behavior of these markers at specific concentrations. Similar behavior was observed up to the stimulation using 50 µg/mL of CAPE. After that, IL-10, E-selectin, and ET-1 may be combined with IL-6 as the next cluster. Moreover, similar to the results for EEP treatment, the behavior of ICAM-1 after CAPE treatment is also disparate from that of the other markers tested.

**Figure 6:**
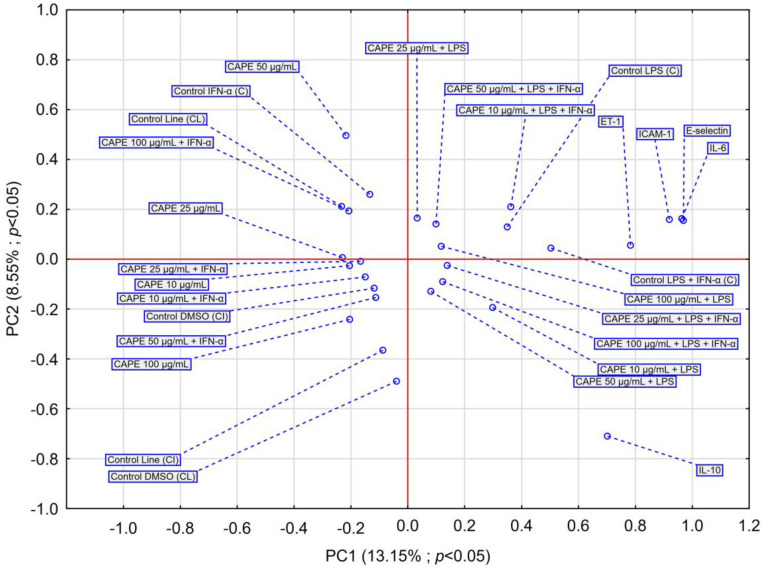
PCA score plot of obtained data based on the effect of CAPE at different concentrations on IL-6, IL-10, E-selectin, ET-1, and ICAM-1 secretion by HGF-1 fibroblasts stimulated by LPS, IFN-α, and LPS + IFN-α.

The PCA obtained for the effect of CAPE on selected markers (Figure 6) indicates a similar effect of CAPE on IL-6, ICAM-1, and E-selectin, but shows a different behavior presented by IL-10. CAPE shows no significant impact on IL-10. In LPS and LPS + IFN-α-induced-HGF-1, the treatment of CAPE exhibits a marked impact on the evaluated markers; however, it is worth noting that CAPE exerts an impact in a dose-dependent manner. At concentrations of 100 µg/mL, CAPE causes a strong impact on markers activated by LPS + IFN-α in HGF-1 culture. Moreover, at a concentration of 50 µg/mL, CAPE demonstrates a strong influence on ET-1.

Based on the obtained PCA results for EEP and CAPE, we can observe that CAPE exhibits a different impact than does EEP.

## 4. Discussion

Periodontal inflammation caused by periodontopathogens in the oral cavity is associated with atherosclerosis and cardiovascular diseases [19]. The most pathogenic periodontal bacteria are Gram-negative anaerobic bacteria from a red-complex, consisting of *Tannerella forsythia*, *Tannerella denticola*, and *Porphyromonas gingivalis* [19,20]. These microorganisms can damage the vascular endothelium through both indirect and direct pathways. The lipopolysaccharides (LPS) of Gram-negative bacteria are widely recognized as initiators of inflammation at the local and systemic levels. LPS interacts with Toll-like receptors, predominantly receptor 4, prompting epithelial cells in the host to discharge pro-inflammatory cytokines [21]. In chronic periodontitis, compared to healthy gingiva, the concentrations of pro-inflammatory cytokines, such as IL-1β, IL-6, TNF-α, and inflammatory mediators, like prostaglandins and metalloproteinases, increase significantly. This can result in the destabilization of the atherosclerotic plaque, which is a major cause of acute coronary syndromes and myocardial infarction [3]. In vitro and in vivo studies have shown that natural compounds, including propolis, possess anti-inflammatory [22], antioxidant [23], anticancer [24], neuroprotective [25], antiviral [26], antibacterial [27], or immunomodulatory properties [28]. The cardioprotective, among others, effect of propolis was confirmed by the scientific team of Oršolić N. et al. [29] and Ji C. et al. in vivo studies [30]. The administration of an ethanolic extract of propolis at a dosage of 50 mg/kg for 30 days demonstrates an anti-atherosclerosis effect in mice treated with a high-fat diet. This effect is achieved by reducing the oxidation of LDL through the activation of the transcription factor NrF2. Additionally, there is an enhancement of the antioxidant enzymes, including phase II detoxification enzymes, hemeoxygenase-1, and enzymes associated with GSH metabolism. Another contributing mechanism involves the elimination of oxidant species, thereby preventing the activation of the NF-κB signalling pathways [29]. Propolis significantly ameliorated the degree of carotid restenosis, inhibited neointima hyperplasia, reduced the serum lipids profile, and enhanced the anti-oxidative activities in hypercholesterolemia rabbits. Propolis led to a decrease in the circulating concentrations of C-reactive protein (CRP), IL-6, TNF-α, and inhibited the expression of TLR4, CD68, NF-κB p65, MMP-9, and TNF-α in the carotid arteries [30].

It is known that inflammation, as a result of periodontitis, increases systemic inflammation and contributes to cardiovascular diseases [3]. In the etiology of ischemic heart disease, inflammatory factors, including both viral and bacterial factors, are considered. The main factor in the inflammatory process is the LPS of Gram-negative bacteria. In addition, viral infections cause the release of IFN-α by cells of the immune system.

The activity of IFN-α is enhanced by LPS, as shown by studies of Parra-Izquierdo I. et al. They have demonstrated that IFN-α acts as a pro-inflammatory and pro-osteogenic cytokine in human aortic valve interstitial cells, and IFN-α is associated with aortic valve calcification [12]. Therefore, the anti-inflammatory effect of propolis and its compound— CAPE—was evaluated in the HGF-1 cell line following LPS and INF-α stimulation. The obtained results indicate that propolis in low concentrations (25 µg/mL) decreases IL-6 in LPS-induced HGF-1. A similar observation was presented by Bueno-Silva B. et al. in which I LPS-activated peritoneal macrophages [31]. Another study of propolis showed that Brazilian ethanol extract of propolis reduces the production of IL-6 in the presence, as well as the absence, of LPS [32].

Additionally, hydroxyethanolic extract of propolis from Italy, at the same concentration as that of Polish propolis (25 µg/mL), significantly reduced IL-6 in LPS-induced human peripheral blood mononuclear cells. Additionally, the isolated compound from propolis—CAPE—at a concentration (25 µg/mL) decreased IL-6, as well as IL-1β, in the conducted study [33].

A similar situation was observed in the case of CAPE in our study. CAPE decreased the level of pro-inflammatory cytokine in LPS and LPS + IFN-α-induced HGF-1.

It is worth noting that patients with coronary artery disease have an elevated level of pro-inflammatory cytokines but may also experience elevated anti-inflammatory cytokines such as IL-10. Regarding our results, EEP at a concentration of 25 µg/mL in LPS + IFN-α-induced HGF-1 increased the level of IL-10. A similar situation was observed by Bueno-Silva in LPS-activated macrophages. Neovestitol from red propolis increased the level of IL-10 [34].

Additionally, geopropolis increased the production of IL-10 in LPS-induced human monocytes [32]. The increase in the production of IL-10 has been observed in LPS stimulated human peripheral blood mononuclear cells when treated with propolis from Morocco [35].

In our study, only EEP, at a concentration of 25 µg/mL in HGF-1-induced by LPS + IFN-α, caused an increase in IL-10. However, we noticed that CAPE, at a range of 10–100 µg/mL, caused an increase in IL-10 following IFN-α stimulation.

However, ethanolic extracts of propolis coming from Malaysia reduced the level of IL-10 in RAW 264.7 macrophages [36].

Interestingly, EEP alone, as well as in combination with IFN-α, resulted in a statistically significant decrease in the IL-10 concentration.

In addition to interleukins, adhesion molecules are also sensitive predictors of inflammation. Moreover, pro-inflammatory cytokines stimulate the release of adhesion molecules, which play an important role in the mechanisms of cardiovascular inflammation and vascular endothelial dysfunction. Adhesion molecules are modern markers of heart failure. Besides vascular cell adhesion molecule 1 (VCAM-1), ICAM-1 is one of the most important molecules in an inflammatory response. ICAM-1, as an endothelial adhesion molecule, stimulates leukocyte adhesion and is involved in penetration into the subendothelial space of the vessels [37].

The results of the study conducted by Jarosz and Nowicka demonstrated that the presence of VCAM-1 and ICAM-1 may indicate the risk of sudden cardiac incidents in patients with documented disease [38].

Based on the literature data, we found that propolis may downregulate the expression of VCAM-1 and ICAM-1 [39].

In the conducted study, we found that EEP treatment showed a noticeable decrease in IFN-α-induced HGF-1. In the case of CAPE, we observed that CAPE, in low concentrations at a range of 10–25 µg/mL, decreased the production of ICAM-1. However, in LPS and LPS + IFN-α-induced HGF-1, treatment downregulated the expression of ICAM-1 in a range of 10–100 µg/mL. Green propolis, whose major compound is artepillin C, resulted in the downregulation of ICAM-1 and VCAM-1 in a concentration of 20 µg/mL [40].

E-selectin (CD62E or ELAM-1) is an endothelial cell surface molecule playing an important role in leukocyte adhesion and rolling leukocytes on the activated endothelium. Its expression is upregulated by pro-inflammatory cytokines, such as IL-1β, IL-6, TNF-α, and lipopolysaccharide. E-selectin plays a significant role in various disorders, such as inflammatory diseases, cardiovascular disorders, metastasis, and cancer [41]. In our experiment, we have shown that only CAPE, at all concentrations, with LPS statistically significantly decreased the concentration of E-selectin in comparison to the results for the control (HGF-1 cell stimulation with LPS). Interestingly, EEP with IFN-α statistically significantly decreased the concentration of E-selectin in comparison to the results for the control (HGF-1 cell stimulation with IFN-α). In our previous work, we have shown that the ethanolic extract of Brazilian green propolis decreased the levels of E-selectin in both tested concentrations (1 μg/mL and 10 μg/mL) [42]. Choi J. S. et al. have demonstrated that TNF-α significantly induced the human umbilical vein endothelial cell (HUVEC) protein expression of adhesion molecules VCAM-1, ICAM-1, and E-selectin under increasing mRNA levels [43]. They noted variations in the effectiveness of different flavonoid subgroups in inhibiting TNF-α-induced monocyte adhesion. Flavones were the most potent flavonoids, while flavanols and flavanones showed no ability to prevent monocyte adherence on TNF-α-activated endothelial cells. Furthermore, they demonstrated that the flavones luteolin and apigenin, at a non-toxic dose, almost completely inhibited the expression of VCAM-1, ICAM-1, and E-selectin proteins. Jeong Y. J. et al. conducted a study aimed at investigating the potential impact of polyphenolic flavonoids on the interaction between monocytes and endothelial cells, as well as the expression of lectin-like oxidized LDL receptor 1 (LOX-1) during the early stages of atherosclerosis development [44]. The results revealed that the flavones luteolin and apigenin hindered the adhesion of THP-1 cells to oxidized LDL-activated human umbilical vein endothelial cells (HUVEC). However, the flavanols epigallocatechin gallate and catechin, the flavonols quercetin and rutin, and the flavanones naringin, naringenin, hesperidin, and hesperetin did not exhibit such inhibitory effects.

Among the adhesion molecules, ET-1 plays a key role in the pathophysiology of chronic heart failure. Among patients who suffer from ischemic heart failure, neurohormonal activation and tissue hypoxia increase the production of ET-1. The mentioned molecule exhibits vasoconstrictor effects and stimulates the proliferation of smooth muscle cells through the activation of ET-1 receptors [45].

Moreover, the disruption in the equilibrium between endothelin (ET) and nitric oxide (NO) contributes to endothelial dysfunction, with ET-1 being a prominent molecular form that plays a crucial role in the development of atherosclerosis. Excessive ET-1 concentrations in the blood cause vasoconstriction, an increase in pressure, and a decrease in organ blood flow. This causes their ischemia and leads to oxidative stress. Endothelin, therefore, plays an important role in the pathogenesis of many diseases, including periodontitis. Scientists indicated that propolis may inhibit endothelin and vascular endothelial growth factor (VEGF) secretion [46]. Chinese propolis, combined with royal jelly and bee venom, reduced the levels of angiotensin II, endothelin-1, and transforming growth factor-β and consequently, led to a hypotensive effect [47]. Our results indicated that propolis at all studied concentrations reduced the level of ET-1 in IFN-α, LPS, and IFN-α + LPS-induced HGF-1. Therefore, EEP seems to be an effective natural product for decreasing ET-1. Regarding the impact of CAPE on the regulation of ET-1, it was observed that it reduced ET-1 in a range of 10–100 µg/mL in IFN-α induced HGF-1. However, in stimulation with LPS and combination LPS + IFN-α, it exhibits a decrease in the level of ET-1 in the highest concentrations in the range of 25–100 µg/mL. It is worth emphasising that CAPE is a major component of European propolis, possessing a vasorelaxant activity.

It seems that propolis and its main ingredient—CAPE—may have a cardioprotective effect by affecting selected predictive factors. Delaprane, J.B., and Abdalla, D.S. [11], emphasized the cardioprotective activity of propolis, and our study confirmed that EEP and CAPE may generate beneficial cardiovascular effects influencing selected factors.

## 5. Conclusions

The association between periodontitis and cardiovascular diseases is evident. Among the mediators that play an important role in the pathomechanism of periodontitis and cardiovascular diseases are interleukins. Adhesion molecules such as E-selectin, ICAM-1, and ET-1 may be modern predictors of coronary artery disease. EEP and the major compound of European propolis—CAPE—influence the expression of cytokines. Based on the conducted study, it is evident that EEP and CAPE may generate beneficial cardiovascular effects. EEP and CAPE exert an impact on cytokines in a dose-dependent manner. Propolis consists of a mixture of many compounds, so its activity results from the combined activities of many complexes, and its action on selected factors is different from that of CAPE. The possibility of using propolis as a cardioprotective agent should also be considered. Elucidating the modulation of cardiovascular disease markers is very important in explaining the mechanisms by which propolis exerts cardioprotective action. It is worth noting that potentially, propolis may have cardioprotective effects by affecting the levels of the interleukins and adhesion molecules, which correlate with changes in the endothelium and the occurrence of coronary artery disease.

## Figures and Tables

**Table 1:** The effect of EEP on the secretion of selected interleukins and adhesion molecules in LPS, IFN-α, and LPS + IFN-α-induced HGF-1 (*n* = 3). Statistical significance was calculated using Fisher’s LSD test. Measured outcomes in bold show statistical significance according to the Fisher’s LSD test. The results of multivariate tests of significance are also shown (F = 10.253, *p* < 0.05).

Sample	IL-6	SD	*p*	IL-10	SD	*p*	E-Selectin	SD	*p*	ICAM-1	SD	*p*	ET-1	SD	*p*
Control line (EI)	37.83	0.00		4.00	0.00		1.01	0.000		3398	0.000		80.28	0.000	
Control DMSO (EI)	29.44	3.29		2.60	0.89		1.87	1.23		3333	230		74.00	6.52	
Control IFN-α (E)	26.90	2.17		1.21	0.69		1.39	0.86		3397	126		49.70	4.08	
EEP 10 µg/mL	20.82	3.88	0.0764	0.87	1.07	**0.0000**	1.67	0.59	0.3026	3269	256	0.8464	50.03	3.03	**0.0000**
EEP 25 µg/mL	20.35	9.12	0.0690	0.22	0.01	**0.0000**	0.82	0.16	0.7711	2670	423	0.2783	32.25	1.27	**0.0000**
EEP 50 µg/mL	32.43	17.94	0.5675	0.20	0.00	**0.0000**	0.91	0.71	0.8849	2557	351	0.2117	18.38	2.19	**0.0000**
EEP 100 µg/mL	29.32	2.09	0.3686	0.20	0.01	**0.0000**	0.27	0.16	0.2517	2116	593	0.0599	14.33	1.63	**0.0000**
EEP 10 µg/mL + IFN-α	62.62	4.56	**0.0000**	0.18	0.01	**0.0006**	0.73	0.74	0.1516	2305	671	**0.0247**	28.81	2.83	**0.0000**
EEP 25 µg/mL + IFN-α	75.45	4.56	**0.0000**	0.19	0.01	**0.0007**	0.18	0.00	**0.0100**	2626	802	0.1074	14.81	1.77	**0.0000**
EEP 50 µg/mL + IFN-α	77.70	5.75	**0.0000**	0.20	0.01	**0.0007**	0.36	0.16	**0.0270**	2699	78.9	0.1441	13.60	1.54	**0.0000**
EEP 100 µg/mL + IFN-α	52.32	9.57	**0.0004**	0.18	0.01	**0.0005**	0.27	0.16	**0.0164**	2000	417	**0.0048**	13.51	1.81	**0.0000**
Control line (EL)	30.99	0.00		0.20	0.00		0.45	0.00		644	0.000		71.33	0.00	
Control DMSO (EL)	26.31	1.06		0.20	0.01		0.46	0.48		2009	115		69.15	0.61	
Control LPS (E)	35.43	4.00		0.20	0.01		0.45	0.48		1737	1001		77.11	1.79	
Control LPS + IFN-α (E)	36.00	10.82		0.19	0.01		1.02	0.97		1612	1059		50.41	6.41	
EEP 10 µg/mL + LPS	38.00	10.14	0.6989	0.20	0.00	0.9972	0.64	0.42	0.6846	2224	1022	0.3059	34.94	7.46	**0.0000**
EEP 25 µg/mL + LPS	21.80	9.28	**0.0459**	0.18	0.01	0.9499	0.55	0.42	0.8388	1184	677	0.2447	26.44	2.03	**0.0000**
EEP 50 µg/mL + LPS	36.37	15.88	0.8872	0.19	0.01	0.9638	0.27	0.16	0.6760	1902	345	0.7273	17.63	1.29	**0.0000**
EEP 100 µg/mL + LPS	40.23	6.91	0.4726	0.19	0.01	0.9805	0.64	0.32	0.6843	2157	185	0.3763	13.87	0.67	**0.0000**
EEP 10 µg/mL + LPS + IFN-α	78.07	5.24	**0.0000**	0.18	0.001	0.9791	0.46	0.48	0.2185	2315	508	0.1413	25.60	2.55	**0.0000**
EEP 25 µg/mL + LPS + IFN-α	80.54	9.89	**0.0000**	0.21	0.00	0.9568	0.55	0.42	0.3001	2740	363	**0.0206**	13.88	0.13	**0.0000**
EEP 50 µg/mL + LPS + IFN-α	85.80	4.39	**0.0000**	0.19	0.01	0.9847	0.64	0.32	0.4032	2455	564	0.0794	13.11	0.72	**0.0000**
EEP 100 µg/mL + LPS + IFN-α	52.04	2.92	**0.0199**	0.19	0.01	0.9944	0.55	0.42	0.3001	2454	601	0.0799	14.60	0.00	**0.0000**

**Table 2:** The effect of CAPE on the secretion of selected interleukins and adhesion molecules in LPS, IFN-α, and LPS + IFN-α-induced HGF-1 (*n* = 3). Statistical significance was calculated using Fisher’s LSD test. The measured outcomes in bold show statistical significance according to the Fisher’s LSD test. The results of multivariate tests of significance are also shown (F = 8.404, *p* < 0.05).

Sample	IL-6	SD	*p*	IL-10	SD	*p*	E-Selectin	SD	*p*	ICAM-1	SD	*p*	ET-1	SD	*p*
Control Line (CI)	68.97	0.00		2.57	0.00		0.02	0.00		3086	0.00		12.26	0.00	
Control DMSO (CI)	94.20	34.70		1.99	0.62		0.22	0.33		3138	121		12.16	0.42	
Control IFN-α (C)	129.5	21.07		1.16	0.21		0.41	0.33		3209	482		12.79	0.52	
CAPE 10 µg/mL	72.42	5.26	0.9704	1.70	0.24	0.1440	0.42	0.69	0.6459	3284	535	0.7092	10.11	0.36	**0.0110**
CAPE 25 µg/mL	93.92	6.35	0.7887	1.57	0.12	0.0947	0.02	0.00	0.9984	3083	288	0.9953	10.29	1.10	**0.0191**
CAPE 50 µg/mL	109.1	12.47	0.6667	0.57	0.10	**0.0014**	0.22	0.33	0.8242	3514	571	0.4233	11.02	0.61	0.1334
CAPE 100 µg/mL	144.0	33.74	0.4218	2.15	0.45	0.4775	0.12	0.16	0.9119	3059	165	0.9588	10.00	0.49	**0.0078**
CAPE 10 µg/mL + IFN-α	133.9	7.78	0.9473	1.84	0.71	0.1097	0.22	0.33	0.7549	2951	847	0.4941	12.03	1.40	0.1937
CAPE 25 µg/mL + IFN-α	152.9	4.95	0.7224	1.69	0.47	0.2043	0.32	0.51	0.8818	3077	502	0.7254	11.50	0.88	**0.0294**
CAPE 50 µg/mL + IFN-α	163.6	13.57	0.6057	2.12	0.65	**0.0253**	0.03	0.00	0.5335	3469	399	0.4907	11.31	0.73	**0.0135**
CAPE 100 µg/mL + IFN-α	210.9	28.17	0.2204	1.24	0.43	0.8563	0.41	0.45	0.9966	3407	695	0.5994	10.30	0.97	**0.0001**
Control Line (CL)	77.52	0.00		1.11	0.00		0.02	0.00		3007	0.00		11.13	0.00	
Control DMSO (CL)	65.22	3.82		2.96	0.21		0.02	0.00		3648	262		11.51	0.33	
Control LPS (C)	2680	200.10		2.49	0.25		9.15	1.47		4626	362		12.79	0.00	
Control LPS + IFN-α (C)	3128	98.89		2.97	0.90		12.24	0.72		4834	179		13.63	0.76	
CAPE 10 µg/mL + LPS	2099	226.5	**0.0000**	3.01	0.16	0.2172	6.81	1.06	**0.0005**	4075	725	0.1487	13.63	0.76	0.1497
CAPE 25 µg/mL + LPS	1444	25.10	**0.0000**	1.75	0.46	0.0762	3.85	0.67	**0.0000**	3547	326	**0.0062**	12.25	0.54	0.3500
CAPE 50 µg/mL + LPS	1392	49.98	**0.0000**	2.47	0.95	0.9511	4.75	1.04	**0.0000**	3618	464	**0.0102**	11.89	0.32	0.1219
CAPE 100 µg/mL + LPS	1618	69.18	**0.0000**	2.20	0.50	0.4757	4.18	0.34	**0.0000**	4304	82	0.3937	11.50	0.88	**0.0287**
CAPE 10 µg/mL + LPS + IFN-α	2716	111.80	**0.0000**	2.31	0.72	0.4166	9.74	0.94	**0.0033**	4335	781	0.4893	13.79	0.84	0.5289
CAPE 25 µg/mL + LPS + IFN-α	1818	40.27	**0.0000**	2.39	0.44	0.1701	5.43	0.86	**0.0000**	4042	110	**0.0403**	11.61	0.44	**0.0010**
CAPE 50 µg/mL + LPS + IFN-α	1808	37.06	**0.0000**	1.95	0.53	**0.0173**	4.53	1.17	**0.0000**	3871	225	**0.0138**	12.08	0.32	**0.0094**
CAPE 100 µg/mL + LPS + IFN-α	1994	63.41	**0.0000**	2.49	0.25	0.2554	6.35	1.44	**0.0000**	3622	300	**0.0024**	11.51	0.33	**0.0006**

## Data Availability

Data are contained within the article.
